# A Critical Comparison of Methods for the Analysis of Indigo in Dyeing Liquors and Effluents

**DOI:** 10.3390/ma7096184

**Published:** 2014-08-29

**Authors:** Valentina Buscio, Martí Crespi, Carmen Gutiérrez-Bouzán

**Affiliations:** Institut d’Investigació Tèxtil i Cooperació Industrial de Terrassa (INTEXTER), Universitat Politècnica de Catalunya (UPC), C/Colom 15, 08222 Terrassa, Spain; E-Mails: valentina.buscio@intexter.upc.edu (V.B.); crespi@etp.upc.edu (M.C.)

**Keywords:** indigo dye, method validation, hexacyanoferrate titration, spectrophotometric, dye baths, effluent

## Abstract

Indigo is one of the most important dyes in the textile industry. The control of the indigo concentration in dyeing liquors and effluents is an important tool to ensure the reproducibility of the dyed fabrics and also to establish the efficiency of the wastewater treatment. In this work, three analytical methods were studied and validated with the aim to select a reliable, fast and automated method for the indigo dye determination. The first method is based on the extraction of the dye, with chloroform, in its oxidized form. The organic solution is measured by Ultraviolet (UV)-visible spectrophotometry at 604 nm. The second method determines the concentration of indigo in its leuco form in aqueous medium by UV-visible spectrophotometry at 407 nm. Finally, in the last method, the concentration of indigo is determined by redox titration with potassium hexacyanoferrate (K_3_(Fe(CN)_6_)). The results indicated that the three methods that we studied met the established acceptance criteria regarding accuracy and precision. However, the third method was considered the most adequate for application on an industrial scale due to its wider work range, which provides a significant advantage over the others.

## 1. Introduction

The denim industry constitutes an important part of the textile sector, with an estimated world production of 10^9^ blue jeans annually [1,2]. The characteristic blue color of denim clothes is due to the indigo dye, which is used in the dyeing of cellulose fibers [3,4]. Indigo is one of the oldest natural dyes, although nowadays, due to the high consumption of denim articles, most of them are dyed with synthetic indigo.

**Figure 1:**
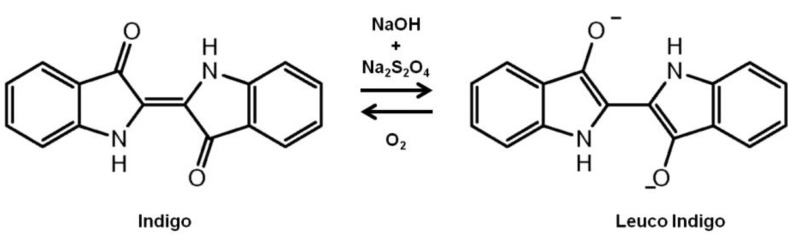
Chemical structure of indigo dye and its leuco form.

Indigo is insoluble in water, but soluble in polar organic solvents. Prior to the dyeing process, it has to be reduced into its leuco form (soluble in water). Currently, the dye reduction step is carried out with sodium dithionite in alkaline medium [3]. The chemical structures of indigo dye and its leuco form (C_16_H_10_N_2_O_2_) are shown in Figure 1.

The main disadvantage of indigo is the fast oxidation of its leuco form in contact with air. For this reason, some of the industrial dyeings are performed in nitrogen atmosphere. In order to ensure the reproducibility and regularity of dyed yarns, it is also important to evaluate the indigo concentration in the dye baths during the dyeing process. The concentration of leuco form should be constant and it is regulated by means of sodium dithionite addition [5].

It is estimated that at least 10 g of indigo are necessary for dyeing one pair of trousers [1,2] and 20% of the dye used is discharged in the wastewater generated during the dyeing process. Based on this information, about two millions of tons are lost in the effluents each year. Several authors have studied the treatment of wastewater that contained indigo dye by either conventional techniques [6] or new proposals such as a stirred tank reactor combined with fixed bed bioreactor [7], electrochemical treatment with boron doped diamond anodes [8] and the use of ferrous oxalate as mediator in a photo-Fenton treatment [9]. In addition, interest in recycling waste products has been extended to indigo-containing effluents. In this way, the U.C.O Company from Belgium [10] and Porter *et al.* [11] studied the recovery of residual indigo from wastewater using ultrafiltration technology.

The aim of the present work was to establish a reliable analytical method for the determination of indigo dye concentration in both the dyeing baths and the residual effluents. In the first case, the indigo must be evaluated during the dyeing process in order to assess the level of dye oxidation. In the case of wastewaters, the analysis of indigo is useful to evaluate the effluent quality and to establish the treatment efficiency.

Since 1967, several studies, summarized in Section 1.1, have been published for the determination of indigo dye [12,13,14,15,16,17]. However, none of them were focused on dyeing baths and effluents control and studies about reliability, reproducibility and accuracy were not included.

### 1.1. Methods for the Analysis of Indigo

As indicated, different analytical methods have been published for the analysis of indigo dye in aqueous media. The main challenge is the low solubility of the dye in water and common organic solvents. Generally, indigo is determined by UV-visible spectrophotometry using different methods that involve treatment of the sample and the addition of organic solvents such as dimethylsulfoxide [12], dichloromethane [13], and pyridine [14]. Indigo has also been determined spectrophotometrically in its leuco form [14] but an acceptable linearity was not obtained due to the fast oxidation of the dye in contact with the air. The solvent addition prevents the indigo dye oxidation in contact with the air and enables the analytical determination by UV-visible spectrophotometry. Amperometric [15] and voltametric [16] methods for the determination of indigo dye have also been reported. Among these methods, Gutierrez-Bouzán *et al.* [14] studied the indigo dye extraction with chloroform and its further determination at 605 nm by UV-visible spectrophotometry. This method is simple and affords good linearity (up to 10 mg·L^−1^).

The use of FIA (Flow Injection Analysis) has also been proposed for the analysis of indigo in its leuco form by Merritt *et al.* [17] as a fast, simple and easy method. However, the main disadvantages of this technique are that it involves a real-time analysis and requires specific equipment. In this study, three different solvents were tested to solubilize the dye: N-methylpyrrolidone (NMP), 2-ethoxyethanol (TEE) and triethanolamine (TEA). Although TEA provided good results, this method required the addition of ferric sulphate to achieve the dye reduction, which causes problems in the dyeing process. Both NMP and TEE solvents were found to be appropriate, but we selected NMP because its structure is rather similar to pyridine, which was successfully tested by Gutierrez *et al.* [12] 

Moreover, Merritt *et al.* [17] investigated the method developed by the company BASF. This method enabled the simultaneous analysis of indigo dye and sodium dithionite, which is the main reducing agent used in the dyeing process. The concentration of both compounds was determined by redox titration using potassium hexacyanoferrate (K_3_(Fe(CN)_6_). The semi-reactions that occur during the titration are shown in Equations (1)–(3):

S_2_O_4_^2−^ + 4OH^−^ → 2SO_3_^2−^ + 2H_2_O + 2e^−^ (1)

C_16_H_10_O_2_N_2_ → C_16_H_10_O_2_N_2_ + 2e^−^ (2)

4Fe(CN)_6_^3−^ + 4e^−^ → 4Fe(CN)_6_^4−^ (3)

The titration curve shows two inflection points. The first point is due to sodium dithionite oxidation in the sample and the second one is attributed to indigo dye oxidation. The indigo concentration is calculated from the difference of titrant solution volume between the two points. This method was also selected and validated in this study due to its simplicity and to the advantage of the double titration. In addition, specific equipment is not required, although it can be used for automation purpose.

In the present work, three methods were studied for the determination of indigo dye in textile effluents in order to select an accurate, fast and easy method, adaptable to industrial processes. The first one is based on the dye extraction with chloroform. The solution is subsequently measured by UV-visible spectrophotometry at its λ_max_ (604 nm). The second one is based on the determination of the indigo in its leuco form by UV-visible spectrophotometry at its λ_max_ (407 nm). In the third method, the concentration of indigo was determined by redox titration using K_3_(Fe(CN)_6_) and an automatic titrator.

## 2. Experimental Procedures

### 2.1. Reagents

Indigo dye (95%) and sodium dithionite (85%) were supplied by ACROS (Sentmenat, Spain). Sodium hydroxide (98.5%) and potassium hexacyanoferrate (99%) were obtained from Panreac (Castellar del Vallès, Spain). Chloroform (99%) and 1-methyl-2-pyrrolidine (99.5%) were purchased from Scharlau (Sentmenat, Spain).

### 2.2. Apparatus

For this study, a Shimadzu UV-visible spectrophotometer UV-2401 (Shimadzu Corporation, Kyoto, Japan) was used for all absorbance measurements. The titration method was carried out in an automatic titrator G20 from Mettler-Toledo equipped with a redox electrode DM 140-SC (Mettler-Toledo, L’Hospitalet de Llobregat, Spain), which is a standard platinum electrode filled with a solution of 3 M potassium chloride and saturated silver chloride. A nitrogen line was installed in order to exclude the oxygen during the titration.

### 2.3. Analytical Methods

Three methods have been selected to determine the indigo dye in dyeing liquors and effluents.

#### 2.3.1. Method 1: Determination of Oxidized Indigo by Chloroform Extraction and UV-Visible Spectrophotometry

A calibration curve was made by dissolving the commercial dye powder in chloroform to obtain five known concentrations. The indigo was not immediately solubilized in chloroform; therefore standard solutions were placed in an ultrasonic bath for 10 min. Complete dissolution of dye was achieved. Then the absorbance values were recorded at λ_max_ = 604 nm.

For determination of indigo in the aqueous samples, an extraction with chloroform was previously performed. Then, the absorbance of the organic phase was measured at λ = 604 nm. The concentration was calculated from the calibration curve.

#### 2.3.2. Method 2: UV-Visible Spectrophotometric Determination of Indigo in Its Leuco form

This method involved the reduction of indigo using the following solution:

(1) 200 mL·L^−1^ 1-methyl-2-pyrrolidine;
(2) 10 g·L^−1^ Na_2_S_2_O_4_;
(3) 13 mL·L^−1^ NaOH 33% (w/v).

The calibration curve was obtained from six known concentrations. The absorbance of the standard solutions was read at the maximum wavelength of the visible spectrum, λ = 407 nm.

The indigo content in the dyeing liquors and effluents was reduced, the absorbance of the solution was measured and the concentration calculated from the calibration curve.

#### 2.3.3. Method 3: Redox Titration

Indigo was determined in its reduced form. The reducing solution used for 1 g of indigo was:

(1) 4 g·L^−1^ Na_2_S_2_O_4_;
(2) 3 mL·L^−1^ NaOH 40% (w/v).

The solution was heated 50 ± 5 °C for 30 min.

The dye should remain in the reduced form for 24 h to ensure the complete reduction reaction. A few drops of anionic dispersant Setamol X-D were added to prevent the aggregation of oxidized indigo molecules. Finally, the sample was titrated with a solution of potassium hexacyanoferrate.

### 2.4. Validation Method

The three methods were validated by means of several parameters [18,19,20,21,22]: Linearity, applicability range, limit of detection (*LoD*), limit of quantification (*LoQ*), accuracy and precision as repeatability and reproducibility.

A wide range of standard solutions of indigo dye (0 to 100 mg·L^−1^) were prepared to establish the range of work. The absorbance was plotted *versus* concentration and only the standards which provide linear correlation were selected to establish the range of work.

The *LoD* and *LoQ* were determined from 10 replicates of standard solution with concentration close to the blank. They were calculated using the following equation [19]:

*LoD* = 3.3 *S*a/*b*; *LoQ* = 10 *S*a/*b* (4)

where *S*a is standard deviation of the standard solution and *b* is the slope of the calibration curve.

For methods 1 and 2 standard solutions of 0.12 mg·L**^−^**^1^ were prepared. For method 3 standard solutions of 50 mg·L^−1^ were prepared.

Accuracy was determined from the recovery of known amounts of indigo (200 and 300 mg·L^−1^) added to an industrial effluent containing 132 mg·L^−1^ indigo dye. Experiments were carried out in duplicate. The recovery percentage was calculated and evaluated.

Repeatability (r) was evaluated from 10 replicates of a standard solution in the same day. The standard solutions were 3.36 mg·L^−1^ for method 1, 4.60 mg·L^−1^ for method 2, and 531 mg·L^−1^ for method 3. The same procedures were carried out on three different days to test reproducibility (R). Repeatability and reproducibility were expressed as relative standard deviations (*RSD*r and *RSD*_R_, respectively).

Finally, the acceptance criteria were established before starting the validation analysis. In this study the acceptance criteria are shown in Table 1.

The theoretical *RSD*r and *RSD*_R_ were calculated using Horwitz equation [23].

**Table 1:** Acceptance criteria established in the validation study.

Parameter	Requirement
Linearity	*R*^2^ > 0.99
Accuracy	Recovery 80%–115%
Repeatability	*RSD*_r_ < 0.5 *RSD*_r_ theoretical
Reproducibility	*RSD*_R_ < 0.5 *RSD*_R_ theoretical

## 3. Results and Discussion

### 3.1. Validation Test

Results of the validation are displayed in Table 2.

**Table 2:** Results obtained in the validation study.

Parameter	Method 1	Method 2	Method 3
Range of linearity (mg·L^−1^)	0–5.0	0–10.0	>16.7
*LoD* (mg·L^−1^)	0.02	0.03	5.5
*LoQ* (mg·L^−1^)	0.08	0.10	16.7
Accuracy (%)	89.4	99.8	83.7
*RSD*_r_ (%)	5.17	1.37	2.65
*RSD*_R_ (%)	0.47	0.44	0.60

According to Table 1, the best results were obtained using method 2, which involved indigo determination in the leuco form using absorption spectroscopy.

Repeatability and reproducibility values obtained in the three studied methods were under 6% and 1% respectively. The repeatability of method 1 was higher than the others. This is probably due to the low solubility of the dye in chloroform.

Regarding the accuracy test, the recovery obtained is shown in Table 3.

**Table 3:** Accuracy obtained for each level of concentration studied.

Sample (mg·L^−1^)	Indigo Added (mg·L^−1^)	Recovery (%)
132	200	Method 1: 96.7
		Method 2: 95.3
		Method 3: 84.2
132	300	Method 1: 82.1
		Method 2: 104.4
		Method 3: 83.4

Although method 3 gave lower dye recovery than methods 1 and 2, it did meet the established acceptance criteria. In method 2, about 100% of recovery was achieved, which demonstrates the high accuracy of this method. Method 3 showed similar recovery for each level of concentration. However, in method 1, when 300 mg·L^−1^ of indigo was added to the sample, the results exhibited lower recovery than when 200 mg·L^−1^ was used. The effect of high indigo dye concentration in results of method 1 is discussed in the Section 3.2.

The lowest range of linearity was obtained in method 1 due to the limited solubility of the indigo dye in chloroform. However, method 3 can only be applied with an indigo concentration above 16.7 mg·L^−1^.

Finally, methods 1 and 2 exhibited similar *LoD* and *LoQ*. It is important to highlight the *LoQ* obtained in method 3. Industrially, about 3–4 g·L^−1^ of indigo dye are used; therefore, this method has an advantage with respect to the other ones: the samples do not need a dilution step. In addition, redox titration does not require a previous preparation of dye baths, because the dye is already in its leuco form due to the addition of sodium dithionite in alkaline medium. Consequently, this method is much faster than the other ones.

### 3.2. Application of the Methods to the Analysis of Effluents and Dyeing Liquors

Five industrial effluents supplied by the denim yarn factory “Tejidos Royo” (Alcudia de Crespins, Valencia, Spain) were selected to evaluate the applicability of the three studied methods.

Sample 1 was the wastewater generated in first washing step after the dyeing process. Samples 2–5 were dye liquors collected during the dyeing process.

The indigo content in the samples was determined at least five times with each method. The average results are shown in Table 4.

**Table 4:** Results from application of methods 1–3 to industrial samples.

Sample	Indigo Dye Concentration (mg·L^−1^)		
	Method 1	Method 2	Method 3
1	117	128	101
2	1300	1390	1290
3	780	940	800
4	1580	3080	3010
5	1700	4160	4060

The indigo concentration in industrial samples was unknown. As the validation tests (Section 3.1) showed high accuracy using method 2, values obtained with this method were selected as the reference levels.

From Table 3, it can be observed that method 2 provided the highest values for indigo dye concentration. With low indigo concentration, method 1 provided similar results to method 2. However, with high indigo concentration, method 3 exhibited similar results to method 2, whereas method 1 should be discarded. These differences are probably due to the high dilution rate, which produced errors in the determination and the low solubility of indigo in chloroform.

**Figure 2:**
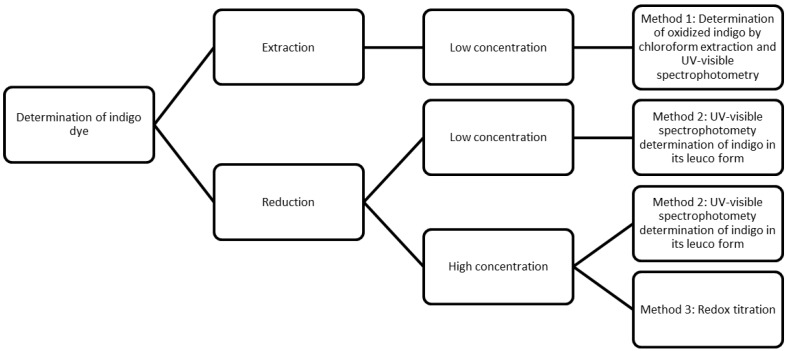
Scheme of the studied methods and their applicability.

In summary, the three studied methods are suitable to be applied in the determination of indigo dye in textile effluents which have lower indigo concentration. In the case of dyeing liquors, methods 2 and 3 are more suitable (Figure 2).

## 4. Conclusions

The validation process carried out in this work showed that all three studied methods can be applied to the determination of indigo dye in both dyeing baths and wastewater effluents.

Method 1 is the most laborious from the experimental point of view because it requires a previous extraction of the indigo in chloroform and a posterior stabilization of the sample in an ultrasonic bath. In addition, its low linearity range, between 0 and 5.0 mg·L^−1^, implies generally a sample dilution before the spectrophotometric analysis. Despite that, the method meets the acceptance criteria.

Method 2, based on the determination of indigo in its leuco form, exhibited the best results in terms of validation parameters. About 100% of recovery is achieved, which indicates the high accuracy of the method. In addition, the concentration range (0–10.0 mg·L^−1^) is higher than in method 1, although the sample still required a dilution for the determination.

Method 3 showed the lower recovery value (84%), but its high range of linearity (from 16.7 mg·L^−1^) provides a significant advantage respect the other two studied methods. Moreover, it enables the simultaneous determination of sodium dithionite and indigo dye concentrations, which are both important to ensure the reproducibility and repeatability of the dyeing process. For these reasons, we consider that method 3 is the most suitable for application on an industrial scale. It is also easy for automation and the installation of an automatic titration provides a fast and simple way to control the dyeing of liquors and efficiency of indigo removal after wastewater treatments.
